# Investigation of social capital and its relationship with emotional adjustment in infertile couples: A cross-sectional study

**DOI:** 10.18502/ijrm.v20i2.10501

**Published:** 2022-03-21

**Authors:** Zeinab Hamzehgardeshi, Fereshteh Kalantari, Fatemeh Bakouei, Siavash Moradi, Sepideh Peyvandi, Maryam Shahidi, Atefe Feizi

**Affiliations:** ^1^Sexual and Reproductive Health Research Center, Mazandaran University of Medical Sciences, Sari, Iran.; ^2^Department of Reproductive Health and Midwifery, Nasibeh Faculty of Nursing and Midwifery, Mazandaran University of Medical Sciences, Sari, Iran.; ^3^Student Research Committee, Nasibeh Faculty of Nursing and Midwifery, Mazandaran University of Medical Sciences, Sari, Iran.; ^4^Infertility and Health Reproductive Research Center, Health Research Institute, Department of Midwifery, Faculty of Nursing and Midwifery, Babol University of Medical Sciences, Babol, Iran.; ^5^Medical Education Development Center, Mazandaran University of Medical Sciences, Sari, Iran.; ^6^Infertility Center, Mazandaran University of Medical Sciences, Sari, Iran.; ^7^Department of Biochemistry, Biophysics and Genetics, Faculty of Medicine, Mazandaran University of Medical Sciences, Sari, Iran.; ^8^Hazrat-e Maryam Fertility Center, Sari, Iran.; ^9^Imam Khomeini Infertility Treatment Center, Sari, Iran.

**Keywords:** Social capital, Emotional adjustment, Infertility.

## Abstract

**Background:**

Infertility is an abnormal event in the life of families and can have various consequences on a personal and social level. Therefore, infertile couples need to manage their emotional responses. Social capital, as one of the social determinants of health, can affect mental health.

**Objective:**

This study was conducted to determine the relationship between social capital and emotional adjustment in infertile couples.

**Materials and Methods:**

This cross-sectional study was conducted from October 2018 to February 2019 with 170 infertile couples visiting infertility centers in Sari, Iran. The data collection instruments included the social capital integrated questionnaire, an emotional adjustment scale and a demographic-reproductive checklist.

**Results:**

All the social capital dimensions, except for the groups and networks dimension, had a score of higher than 50 (more than the mean score). Based on ANCOVA and the multiple linear regression results, the dimension of trust and solidarity had a significant negative relationship with emotional adjustment (p = 0.01), but no significant relationship was observed between the other social capital dimensions and emotional adjustment.

**Conclusion:**

The trust and solidarity dimension had a significant relationship with emotional adjustment in infertile couples. Accordingly, increasing mutual trust between neighborhood residents can strengthen social capital, and in turn, improve emotional adjustment in infertile couples.

## 1. Introduction

Infertility has been described as a global health problem with physical, psychological and social consequences (1). It is estimated that between 8% and 12% of couples of reproductive age are infertile (2). The prevalence of infertility in Iran ranges from 10.3%-24.9% (3). Infertility is an abnormal life event and the quality of life of infertile couples can be severely affected by medical interventions (4). Infertile individuals often do not easily adjust to the problems associated with infertility and may be worried about their body and sexual dysfunctions. Emotional adjustment is described as the management of behavioral, mental and emotional responses to infertility. Emotional adjustment does not mean that couples are reluctant to have children or that they have accepted their current situation, but rather it shows the extent to which couples are able to effectively control their own emotional and cognitive processes and how they prepare themselves for both conditions of having or not having a child (5). Cognitive factors, social support and personality traits influence emotional adjustment to infertility (6).

Social capital is described as the resources achieved through social relations (7). Social capital facilitates human interactions and is effective in various aspects of life, including mental health (8). Social capital can support individuals in phases of acute depression and stress (9). According to the World Bank, social capital is created through the impact of social institutions, human relations and norms on the extent and depth of social interactions (10). Social capital has a positive effect on health through mechanisms such as easier access to the information of community members, better health-related decisions, higher adherence to social norms, greater access to and use of health services and more access to mental health services (11). A previous study reported that some structural dimensions of social capital such as groups and networks, and trust and solidarity can lead to social cohesion and inclusion. These dimensions can have a significant association with self-rated health in women (12).

Infertile couples may need to tolerate high stress levels, and they need to manage their behavioral, mental and emotional responses to infertility. Social capital is one of the social determinants of health (13). The need for research in a sample of Iranian infertile couples was identified, given the lack of previous related research in this population. Thus, the present study was conducted to determine the level of social capital and its relationship with emotional adjustment in infertile couples visiting infertility centers in Sari, Iran, during 2018.

## 2. Materials and Methods

This was a cross-sectional study. The sample size needed was calculated to be 310 based on a previous study (8). Given that the score of trust and solidarity dimension of social capital had a standard deviation of approximately 18 and a confidence interval of 4, at the confidence level of 95% and power of 90% and assuming a maximum attrition rate of 10%, the number of participants needed was estimated to be 345. Therefore, 170 infertile couples were studied in this research. Sampling was performed from October 2018 to February 2019 using the convenience sampling method at Imam Khomeini and Hazrat-e Maryam infertility centers in Sari, Iran.

Prior to the initiation of the study, the objectives were fully explained to the participants. They were reassured that their information would be kept confidential and that they could leave the study at any time.

The inclusion criteria included infertility diagnosed by a relevant specialist, the initiation of assisted reproductive methods, willingness to participate in the study, literacy, Iranian nationality and Persian language. The study exclusion criteria comprised of systemic diseases such as diabetes, hypertension, hyperlipidemia or thyroid disorders based on treatment history, having major psychiatric disorders during the study, use of psychiatric medications, tragic events in the past six months like the death of a loved one that had severely affected the couple's mood, addiction to alcohol or drugs based on self-report and unwillingness to continue participation.

Research questions:

1. What is the extent of social capital and its dimensions in infertile couples visiting infertility centers in Sari in 2018?

2. What is the level of emotional adjustment in these infertile couples?

3. What are the socio-demographic factors related to this emotional adjustment?

4. Is there a correlation between the levels of social capital and emotional adjustment in infertile couples visiting infertility centers in Sari during 2018?

### Data collection tools

####  Demographic-reproductive checklist

This checklist included items on personal and family characteristics and information on infertility and treatment. Personal and family characteristics included the age, education and employment status of the woman and her spouse, the age of marriage, the woman and spouse's previous marriage history and the number of children. The infertility information comprised of infertility type, infertility cause, type of treatment, number of treatments and desire to continue treatment.

#### Social capital: An integrated questionnaire (14)

This questionnaire was developed by the World Bank to evaluate social capital in developing countries. The social capital: An integrated questionnaire includes six dimensions of 1) information and communication, 2) groups and networks, 3) trust and solidarity, 4) empowerment and political action, 5) collective action and cooperation, and 6) social cohesion and inclusion. The mean score of five dimensions (excluding the dimension of information and communication which is descriptively stated) is within the range of 0-100, with a higher score indicating higher social capital (15). The validity and reliability of this questionnaire in Iran have been investigated through translation and re-translation (14). Since no study was found on social capital among infertile couples, the research team investigated the reliability of the Persian version of this questionnaire in infertile couples. The Cronbach's alpha coefficient in women was 0.83 and 0.95 in men. Given that a Cronbach's alpha coefficient above 0.7 is described as a good correlation coefficient (16), the questionnaire has good reliability.

#### Emotional adjustment scale (5)

This scale consists of 12 questions and is scored based on a 6-point Likert-type scale ranging from totally agree to totally disagree (6 and 1, respectively) (17). The validity and reliability of this scale in Iran have been investigated through translation and retranslation using quantitative and qualitative indices. The Persian version of the tool was prepared with 10 questions. The minimum score is 10 and the maximum score is 60. A high score means a low adjustment level, and the Cronbach's alpha value is 0.68 (5).

### Ethical considerations

This study was approved by the Ethics Committee of Mazandaran University of Medical Sciences, Sari, Iran (Code: IR.MAZUMS.REC.1398-040). All ethical principles were followed by the authors of this manuscript. Written informed consent was obtained from all participants.

### Statistical analysis

The normal distribution of the variables was assessed using the Kolmogorov-Smirnov test. Then, the quantitative data were described by calculating the mean and standard deviation and/or median and inter-quartile range and the qualitative data were described by frequency and percentage. Correlation was also analyzed by using the Pearson and Spearman correlation coefficients, depending on the type of data distribution. Correlation test, linear regression and ANCOVA were used to adjust the socio-demographic factors related to the level of emotional adjustment. The data were analyzed using IBM's Statistical Package for the Social Sciences (SPSS) version 21 (USA), and in all the statistical tests, a two-way p-value of less than 0.05 was considered significant.

## 3. Results

The age range of women varied from 19-42 yr and men from 25-53 yr. The maximum length of infertility was 17 yr and the minimum was one yr. The most frequent level of education was pre-university or diploma for both men and women. Most women were housewives, and most men were self-employed. The most frequent type of treatment was herbal medicine with 54.1% and the least frequent was intracytoplasmic sperm injection with 0.6%. In addition, 74.7% of the couples had primary infertility and 25.3% had secondary infertility. The most common cause of infertility was unknown (43.8%), and 99.4% of the couples wanted to continue treatment.

The basic characteristics, the status of the social capital dimensions and the emotional adjustment scores of the participants are shown in table I. The scores of emotional adjustment and dimensions of social capital of the participants are presented in table II; Pearson correlation coefficients between the dimensions of social capital and emotional adjustment are displayed in table III; and the results of linear regression of emotional adjustment are exhibited in table IV.

In infertile women, no significant difference was found among women with different levels of education with respect to the social capital dimensions, but among infertile men with different levels of education, a significant difference was noted in the dimensions of trust and solidarity (p = 0.02) and collective action and cooperation (p = 0.02). In infertile women, a significant difference was observed among women of different occupations with regard to the empowerment and political action dimension (p = 0.04), and in men a statistically significant difference was observed in those of different occupations in the dimension of groups and networks (p = 0.04).

A difference in the score of emotional adjustment was found among women with different levels of education, although this was not statistically significant (p = 0.08). Also, the difference in the score of emotional adjustment among men with different levels of education was not significant. The emotional adjustment score in infertile men and women of different occupations showed a significant difference (Table I).

The score of emotional adjustment was higher in women than in men, and this difference was significant (p = 0.01). Due to the fact that a higher score is a sign of lower adjustment, in general, emotional adjustment was less in women than in men. It should be noted that the minimum score of emotional adjustment was 10 and the maximum score was 60. Emotional adjustment scores were above average in both women and men. Social capital was also higher in men than in women in all of the dimensions, but only the dimensions of collective action and cooperation (p = 0.03) and empowerment and political action (p < 0.001) had statistically significant values.

Among all the dimensions of social capital, only the two dimensions of groups and networks, and trust and solidarity had a negative relationship with emotional adjustment. Almost all the dimensions of social capital were directly and significantly correlated with each other. The exceptions were that a significant correlation was not observed between the dimensions of groups and networks, and social cohesion and inclusion.

The socio-demographic variables and the dimensions of social capital as the independent variables, and emotional adjustment in infertile couples as the dependent variable, were entered into a linear regression model to examine their adapted relationship. A significant inverse relationship was found between trust and solidarity, and emotional adjustment (p = 0.01).

**Table 1:** Basic characteristics, status of social capital dimensions and emotional adjustment scores of n = 340 participants


**Variable**		**Frequency (%)**	**Groups and networks**	**Trust and solidarity**	**Collective action and cooperation**	**Social cohesion and inclusion**	**Empowerment and political action**	**Emotional adjustment mean (SD)**
**Woman's education**								
	**Primary school**	7 (4.1)	44.70 ± 15.73	62.86 ± 22.97	60.71 ± 17.81	75.86 ± 8.03	61.69 ± 19.52	40.86 ± 3.53
	**Elementary** ** school**	8 (4.7)	36.76 ± 11.93	55.83 ± 25.15	67.71 ± 24.57	76.45 ± 6.81	57.61 ± 12.00	35.13 ± 10.38
	**High school**	16 (9.4)	40.30 ± 20.16	53.64 ± 19.22	57.29 ± 26.33	72.60 ± 9.61	57.46 ± 15.96	41.93 ± 6.87
	**Pre-university** ** or diploma**	72 (42.4)	41.11 ± 18.01	56.69 ± 1.78	55.55 ± 22.02	71.35 ± 10.28	53.68 ± 14.11	39.92 ± 7.10
	**BA**	63 (37.1)	40.77 ± 19.25	57.11 ± 20.59	52.38 ± 24.84	71.03 ± 10.87	52.49 ± 16.50	38.69 ± 7.66
	**MA**	4 (2.4)	38.08 ± 27.11	60.66 ± 7.20	60.41 ± 38.11	70.60 ± 3.81	56.91 ± 11.30	39.75 ± 8.34
	**p-value**	0.44*	0.28*	0.67*	0.14*	0.20*	**0.08
**Man's education**								
	**Primary school**	6 (3.5)	48.37 ± 16.51	51.66 ± 37.58	59.72 ± 11.07	68.05 ± 16.52	60.59 ± 19.79	37.50 ± 7.71
	**Elementary** ** school**	14 (8.2)	39.03 ± 21.38	49.64 ± 21.11	52.38 ± 16.15	73.28 ± 9.05	58.40 ± 12.35	38.21 ± 7.35
	**High school**	22 (12.9)	43.51 ± 18.32	62.18 ± 14.86	61.74 ± 25.15	75.38 ± 7.45	60.95 ± 13.98	40.05 ± 9.44
	**Pre-university** ** or diploma**	73 (42.9)	40.45 ± 19.38	56.94 ± 19.10	60.50 ± 24.76	73.20 ± 10.31	56.33 ± 11.43	36.75 ± 7.32
	**BA**	42 (24.7)	45.57 ± 18.15	59.80 ± 21.59	63.69 ± 21.68	72.91 ± 11.00	59.71 ± 15.05	37.33 ± 8.33
	**MA**	12 (7.1)	46.55 ± 24.84	55.83 ± 23.99	63.19 ± 22.03	77.85 ± 8.74	64.85 ± 13.30	35.83 ± 7.73
	**PhD**	1 (0.6)	49.46 ± 0.0	15.00 ± 0.0	66.66 ± 0.0	75.00 ± 0.0	57.14 ± 0.0	46.00 ± 0.0
	**p-value**	0.33*	0.02*	0.02*	0.16*	0.11*	**0.22
**Woman's job**								
	**Housewife**	142 (83.5)	40.42 ± 18.49	56.74 ± 19.26	46.54 ± 24.16	71.22 ± 10.28	53.42 ± 14.77	39.71 ± 6.75
	**Employee**	13 (7.6)	42.61 ± 22.64	57.43 ± 19.96	55.13 ± 19.70	71.58 ± 10.63	61.28 ± 17.16	34.38 ± 8.98
	**Self-employed**	14 (8.2)	42.69 ± 14.11	58.09 ± 20.43	67.26 ± 21.79	77.31 ± 6.78	54.30 ± 17.69	42.57 ± 9.18
	**Workwoman**	1 (0.6)	41.73 ± 0.0	55.00 ± 0.0	33.33 ± 0.0	72.22 ± 0.0	70.36 ± 0.0	23.00 ± 0.0
	**p-value**	0.69*	0.36*	0.49*	0.23*	< 0.05*	** < 0.001
**Man's job**								
	**Unemployed**	3 (1.8)	40.22 ± 15.15	49.44 ± 23.82	55.55 ± 24.05	76.69 ± 10.86	60.59 ± 8.06	37.50 ± 14.84
	**Employee**	36 (21.3)	48.58 ± 19.58	57.77 ± 22.19	60.88 ± 22.61	75.16 ± 10.20	60.82 ± 14.44	35.28 ± 7.43
	**Self-employed**	93 (55.0)	41.06 ± 18.05	58.46 ± 20.13	62.36 ± 22.34	74.01 ± 9.74	58.73 ± 12.70	38.63 ± 7.96
	**Workman**	32 (18.9)	39.40 ± 22.06	54.83 ± 18.00	57.55 ± 23.60	70.22 ± 11.35	57.38 ± 14.83	36.88 ± 7.82
	**Farmer**	6 (3.0)	53.92 ± 19.63	50.66 ± 27.95	53.33 ± 26.74	72.77 ± 15.22	66.28 ± 4.05	34.60 ± 7.57
	**p-value**	< 0.05*	0.43*	0.28*	0.29*	0.24*	** < 0.01
Data presented as Mean ± SD. Kruskal-Wallis test was used to examine the social capital dimensions by education and occupation. One way ANOVA was used to examine emotional adjustment by education and occupation. BA: Bachelor's degree, MA: Master's degree, PhD: Doctor of Philosophy, *Kruskal-Wallis test, **One Way ANOVA								

**Table 2:** Emotional adjustment scores and dimensions of social capital of n = 340 participants


**Dimensions of social capital**	**Couple**	**Woman**	**Man**	**p-value**
**Groups and networks**	41.77 ± 18.85	40.78 ± 18.38	42.76 ± 19.31	*0.25
**Trust and solidarity**	57.05 ± 19.74	56.89 ± 19.24	57.21 ± 20.29	*0.59
**Collective action and cooperation**	58.21 ± 23.41	55.44 ± 23.80	60.98 ± 22.76	*0.03
**Social cohesion and inclusion**	72.66 ± 10.18	71.75 ± 10.13	73.58 ± 10.19	*0.06
**Empowerment and political action**	56.45 ± 14.45	54.20 ± 15.26	58.69 ± 13.26	*0.01
**Emotional adjustment **	38.44 ± 7.71	39.44 ± 7.41	37.46 ± 7.90	**0.02
Data presented as Mean ± SD. Mann-Whitney U Test was used to analyze dimensions of social capital. Independent *t* test was used to analyze emotional adjustment scores. *Mann-Whitney U test, **Independent *t* test				

**Table 3:** Pearson correlation between the dimensions of social capital and with emotional adjustment


	**Emotional adjustment**	**Groups and networks**	**Trust and solidarity**	**Collective action and cooperation**	**Social cohesion and inclusion**	**Empowerment and political action**
**Groups and networks**	*-0.127	1	- -	- -
**Trust and solidarity**	*-0.132	*0.181	1	- -	-
**Collective action and** ** cooperation**	0.014	*0.117	*0.378	1	- -
**Social cohesion and inclusion**	-0.015	0.073	*0.230	*0.219	1	-
**Empowerment and political** ** action**	-0.054	*0.127	*0.287	*0.400	*0.359	1
**Emotional adjustment **	1	*-0.127	*-0.132	0.014	-0.015	-0.054
Pearson correlation coefficient was used to investigate the relationship between the dimensions of social capital, and with emotional adjustment. *P < 0.001						

**Table 4:** Results of the linear regression of emotional adjustment of n = 340 participants


**Model**	**Unstandardized coefficients B**	**p-value**
**Constant**	41.063	< 0.001
**Groups and networks**	-0.034	0.14
**Trust and solidarity**	-0.061	0.01
**Collective action and cooperation**	0.037	0.07
**Social cohesion and inclusion**	-0.011	0.80
**Empowerment and political action**	-0.017	0.61
**Marriage duration**	0.219	0.16
**Married date**	2.737	0.14
**Duration of infertility**	-0.356	0.06
**Number of medical treatments**	-0.543	0.06
**Number of IUI treatments**	0.249	0.69
**Number of ICSI treatments**	1.972	0.45
**Number of IVF treatments**	-1.383	0.19
IUI: Intrauterine insemination, ICSI: Intracytoplasmic sperm injection, IVF: In vitro fertilization		

## 4. Discussion

In the present study, social capital in infertile couples and its relationship with emotional adjustment were assessed. The results showed that social capital in infertile couples in all the dimensions was higher than 50, except for the dimension of groups and networks. In other words, social capital was above average in most dimensions. The study results were consistent with the findings of a previous study where the score of social capital in all the dimensions was reported above 50, except for the dimension of groups and networks where the score was reported as 18.9 (14). Also, in another study the score of social capital in all the dimensions was higher than 49, except for groups and networks which was reported as 19.6 (18). One-third of infertile men and women try to avoid social contact with others and have less participation in social ceremonies and places by choosing a moderate or high level of isolation strategy (19), which may explain the lower score of infertile couples in the groups and networks dimension.

The results also showed that social capital in the two dimensions of collective action and cooperation, and empowerment and political action was significantly higher in men than in women (20, 21, 13). This finding could be due to the fact that more men than women join the workforce. Gender also affects membership in associations, such that women are more likely than men to become educators or members of charitable services and cultural associations, while men are more interested in working in political associations (22). Therefore, women have less access to valuable resources of social capital than men do.

Another finding was that the score of emotional adjustment in men was lower than that of women. Since a lower score is a sign of greater adjustment, emotional adjustment in women was lower than in men. It has been shown that infertile women experience more stress compared to infertile men (23), depression and anxiety in infertile women may be higher than in their husbands, and their self-esteem may be lower (24). In addition, infertile women may be more prone to isolating from social relationships to reduce the negative consequences of infertility. This may be due to the fact that women are more stressed than men, regardless of the cause of infertility (19), which may be due to social pressure to become a mother. The stress that infertile women endure is similar to that of individuals with cancer, hypertension or AIDS, and most infertility treatments are performed on women (25, 26). This stress can be the reason for women having less emotional adjustment than men. However, a number of studies are inconsistent with the above results, and have shown no significant difference between the adaptability of infertile women and men (27, 28). Therefore, further studies are needed in this area.

Another result of this study was that the dimension of groups and networks showed no significant relationship with emotional adjustment in infertile couples, which is inconsistent with the results of another study that concluded that students in a social structure with higher social capital (mutual trust, social, psychological and financial support, the existence of kinship networks, a sense of belonging and cooperation) had higher mental health (29). The reason for this inconsistency may be differences in the populations.

We found that the dimension of trust and solidarity had a significant negative relationship with emotional adjustment in infertile couples. This finding is inconsistent with the results of other studies that have reported a significant positive relationship between mental health and the components of individual trust and support of social capital (8, 30). This discrepancy could be due to the fact that promoting trust between individuals and thus expanding one's social range and relationships reduces mental pressure, and individuals with higher trust have higher knowledge and skills, and thus, better physical and mental health (31).

This study showed no significant relationship between the dimension of collective action and cooperation, and emotional adjustment in infertile couples, while several studies have concluded contrary to the above results. These have indicated that general social capital creates a kind of mental security and comfort in female heads of household, the result of which is improved mental health (32), and also that social cooperation can have a significant relationship with mental health (33) and, through social cooperation and trust, has been shown to explain 0.59% of social health (34). The probable cause of this difference could be the differences in the populations; thus, further studies in this field are recommended.

We did not find a significant relationship between the dimension of social cohesion and emotional adjustment in infertile couples, which is inconsistent with the results of another study which reported a significant positive relationship between social cohesion and mental health (35). One of the important consequences of social cohesion is inclusion in a group to achieve a goal, in which the group agrees more on the issues and phenomena, thus increasing efficiency and productivity. In such groups, members work better with peace of mind and psychological security as their hope for success increases (36). The reason for this inconsistency in findings could be the correlation between the dimensions of social cohesion, and trust and solidarity, that is, the effect on emotional adjustment is probably indirect through the dimension of trust and solidarity.

Our findings also revealed that the dimension of empowerment and political action had no significant relationship with the emotional adjustment score in infertile couples. Empowerment is the process by which the community becomes aware of its own needs and gains a kind of self-confidence and self-reliance to meet those needs, and accordingly, it gains the necessary ability to achieve its goals. Empowerment includes the five dimensions of self-efficacy, self-determination, personal acceptance of the outcome (feeling of effectiveness), sense of meaning (sense of worth) and trust in others (feeling secure) (37). The perception of social support and a sense of worth will lead to the health of individuals and their mutual responsibilities to the community (34). Given that trust is one of the components of empowerment, it seems that empowerment is effective through this dimension. To the best of the author's knowledge, no study has yet addressed this issue.

## 5. Conclusion

Since the dimension of trust and solidarity had a significant relationship with emotional adjustment in infertile couples, it seems that in Iran, trust in government-run institutions plays a substantial role in building trust and promoting social capital. Thus, the government should provide grounds to increase the presence of individuals in vibrant communities. Formation of various local associations, sports groups, membership of religious and charitable groups, strengthening the spirit of cooperation among individuals and increasing mutual trust between residents of neighborhoods can strengthen social capital, which in turn, can promote the mental health of individuals, especially infertile couples.

##  Conflicts of Interest

The authors declare that there is no conflict of interest.
